# From Digital Engagement to Physical Activity: A Four-Wave Test of Behavioral Spillover in Online Fitness Communities

**DOI:** 10.3390/bs16091645

**Published:** 2026-09-14

**Authors:** Qiuhan Zhu, Youzenan Tu, Xiangnan Li, Pengcheng Chang

**Affiliations:** 1 School of Kinesiology and Physical Education, Zhengzhou University, 100 Kexue Avenue, Zhengzhou 450001, China; zhuqiuhan@zzu.edu.cn (Q.Z.);; 2 School of Management, Zhengzhou University, 100 Kexue Avenue, Zhengzhou 450001, China

**Keywords:** online fitness community, perceived interaction, continuance intention, physical activity, behavioral spillover, longitudinal study

## Abstract

Continued use of fitness technology is often treated as a prerequisite for health behavior change, yet platform engagement and behavioral regulation may operate through different mechanisms. This four-wave panel study tested whether an engagement pathway in online fitness communities extends across the digital–physical boundary. After a priori quality screening, 506 Chinese adults who had used the community functions of a smart fitness platform, application, or wearable device within the previous three months and had access to a community activity venue were retained at T1, with 417 at T2, 377 at T3, and 394 at the outcome-only T4 wave. A unified full-information maximum-likelihood path system adjusted for prior outcomes and demographics and used 2000 participant-level bootstrap resamples. Perceived interaction at T1 predicted social presence (β = 0.297), flow (β = 0.355), and belonging (β = 0.245) at T2, and all three independently predicted continuance intention at T3 (βs = 0.203–0.302). Continuance intention is the intention to keep using the community, not observed use. It showed no detectable association with physical activity at T4 once prior activity was controlled (β = 0.016, 95% CI [−0.061, 0.082]), and the serial indirect effects were likewise undetectable. This did not follow from conditioning on the strong autoregressive path: the zero-order correlation between continuance intention and later activity was 0.068, while associations along the hypothesized within-platform chain ranged from 0.218 to 0.563. Missing-data, complete-case, log-transformed, and inverse-probability-weighted specifications converged. Exploratory equivalence testing rendered standardized effects of ±0.10 or larger implausible (*p* = 0.012) but not ±0.05, and a BIC approximation favored the model without the path (BF_01_ = 17.81). Experiences that sustain digital continuity should not be assumed to produce physical activity spillover.

## 1. Introduction

A 2025 systematic review of continued usage of mobile fitness applications opens from a premise that the field has largely stopped examining: mobile fitness applications can change behavior toward a healthier lifestyle, and “for such a change, the continued usage of mobile fitness applications is a necessary prerequisite, exceeding merely installing and short-term usage of such apps” (M. Chen et al., 2025). The premise is stated as the starting point of the argument rather than as a claim under test.

It is a reasonable premise. A technology that has been abandoned cannot deliver repeated support. Yet it merges two outcomes that are worth keeping apart. Returning to a community, reading posts, exchanging messages, and checking rankings are digital behaviors. Exercising requires bodily effort, time, access, and the regulation of action. Digital continuity creates the opportunity for influence; whether it converts into physical activity is a separate empirical question.

That question has largely gone unasked. Research published in this area shows that technological functions, social comparison, support networks, leaderboard position, and community participation matter for fitness app use and for activity (Huang & Ren, 2020; Huang et al., 2022; H. M. Kim, 2022; Li & Meng, 2023; Yang & Koenigstorfer, 2025), and that motives for persistence differ between novice and experienced users (Stragier et al., 2016). Reciprocal associations among online socializing, physical activity, and emotional states have also been documented (Wang et al., 2025). In the wider literature on fitness applications, however, the dependent variable usually stops at continued usage or the intention to continue. Two recent prospective studies do carry the question further, and both report that the transition succeeds. A two-stage field study built around an app-based running event finds that initial use raises affective and continuance commitment, that these promote continued use in the following period, and that continued use is in turn associated with better subsequent exercise performance (Yao & Chen, 2026). A two-wave study of 4465 respondents with a six-month follow-up finds that app usage styles predict later physical activity levels (Takano et al., 2026).

Both studies measure use. The present study asks about a different quantity: not whether people who keep using an application are more active later, but whether the *intention to continue*—the endpoint at which the technology-continuance literature conventionally stops, and the quantity that engagement design actually delivers—carries across the same boundary once prior activity is held constant. That is the gap this study addresses.

### 1.1. Two Mechanisms with Different Destinations

We distinguish an *engagement-generating* mechanism from a *behavior-regulation* mechanism. The first makes a platform socially vivid, absorbing, and identity-relevant, which sustains the intention to keep using it. The second links motivation to action through self-monitoring, feedback, goal setting, planning, personalization, and prompts. The distinction is theoretical: this observational design estimates associations along the first of the two and identifies neither causally, so *pathway* refers throughout to what was measured here.

Evidence on the second mechanism is substantial and quantified. A meta-analysis of smartphone applications and activity trackers, where the intervention is defined by automated and continuous self-monitoring and feedback, found small-to-moderate increases in physical activity (Laranjo et al., 2021). An umbrella review of 39 systematic reviews and meta-analyses covering 163,992 participants reported standardized mean differences of 0.3 to 0.6 for activity, corresponding to roughly 1800 additional steps and 40 additional minutes of walking per day, with benefits sustained over time (Ferguson et al., 2022).

**Figure 1:**
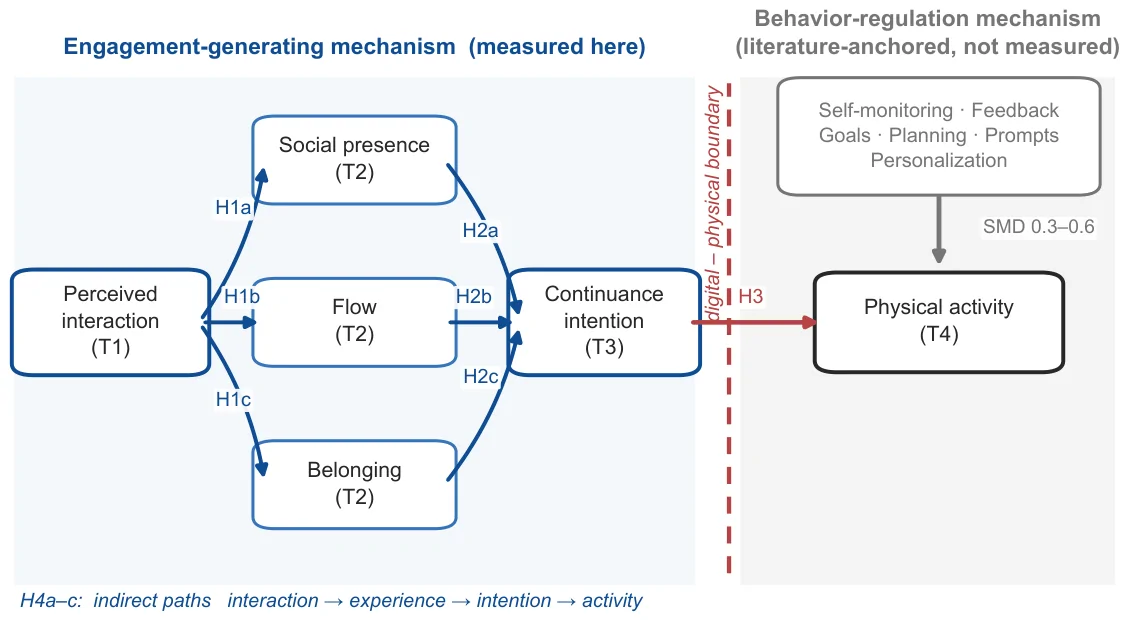
Two mechanisms, one boundary: the hypothesized system. The engagement-generating pathway (blue, **left**) is measured in this study: perceived interaction at T1 is expected to predict social presence, flow, and belonging at T2 (H1a–c), each of which is expected to predict continuance intention at T3 (H2a–c), which in turn is expected to predict physical activity at T4 (H3), with serial indirect paths (H4a–c). The behavior-regulation pathway (grey, **right**) is *not measured here*: continuance intention is the intention to keep using the community, not observed use. Its components are shown as a proposed organizing concept drawn from randomized and meta-analytic evidence (Ferguson et al., 2022; Laranjo et al., 2021), not as observed variables in the present design. The dashed red line marks the digital–physical boundary.

These findings are not evidence that social immersion is ineffective, and the present study does not compare feature classes. They establish something more specific and more useful as an interpretive anchor: digital tools can move physical activity when their design supplies action-regulation functions. A null result for the engagement pathway therefore cannot be read as “digital health tools do not work.” It can only be read as a statement about which pathway carries the effect. Figure 1 shows the separation.

### 1.2. Perceived Interaction as a Bundle of Designed Affordances

Perceived interaction is operationalized as a composite of modality richness, bidirectionality, and response immediacy. Richness captures the variety of cues through which members communicate, bidirectionality captures reciprocal exchange, and immediacy captures the perceived speed of response (Daft & Lengel, 1986; Y. Liu, 2003; S. J. McMillan & Hwang, 2002). We treat these as complementary components that jointly form an interaction index, not as interchangeable manifestations of a reflective second-order factor. Changing one affordance can move the index without requiring the others to move. This resolves the ambiguity that arises when technically distinct affordances are forced into a single reflective measurement model.

### 1.3. Three Platform-Domain Experiences

Social presence is the felt salience of other people in mediated communication (Gefen & Straub, 2003; Short et al., 1976). Flow is focused absorption and intrinsic enjoyment during an activity (Csikszentmihalyi, 1990; Hoffman & Novak, 1996; Jackson et al., 2008). Belonging is the experience of membership, attachment, and identification with a community (Blanchard & Markus, 2004; D. W. McMillan & Chavis, 1986). Each supplies a distinct reason to remain: other members feel present, use feels absorbing, or the community becomes part of the user’s social identity. Expectation-confirmation theory explains why satisfactory post-adoption experiences sustain continued system use (Bhattacherjee, 2001); we borrow that logic to motivate platform continuity and do not claim to test the full model, because confirmation and perceived usefulness were not measured.

### 1.4. From Digital Continuity to Behavioral Spillover

Continuance intention concerns future community use. It is not an intention to exercise. The focal question is therefore not the familiar intention–behavior gap within one behavioral domain. It is a question of cross-domain spillover: does an intention directed at a platform prospectively relate to a bodily health behavior once prior activity is controlled?

Behavioral spillover has been studied mainly across behaviors within a domain, where performing one behavior raises or lowers the likelihood of a related one. Spillover across the boundary between a digital domain and a physical one has rarely been formalized in research on online fitness communities, even though the assumption that it occurs underlies much of the applied rationale for community features. Treating digital continuity and physical activity as distinct outcomes, and testing the crossing rather than assuming it, is the conceptual step this study takes.

Two lines of literature give reason to treat the expected positive crossing as provisional. First, intentions predict behavior less well as the behavior becomes habitual. Past behavior contributes variance to future behavior beyond intention, particularly for behaviors performed repeatedly in stable contexts (Ajzen, 2002; Ouellette & Wood, 1998). In systems research, the same boundary is well documented: habit limits the predictive power of intention for continued use (Limayem et al., 2007), and automatic and reasoned routes to use are empirically separable (S. S. Kim et al., 2005). Second, exercise for established users is precisely the kind of behavior that becomes habitual and context-bound. If physical activity in this population is carried largely by habit, a platform-directed intention has little room to move it. This reasoning is stated here, before the results, because it is the account we invoke later to interpret them.

### 1.5. The Present Study

We tested the engagement pathway and its downstream boundary in a four-wave panel of Chinese adults using the community functions of smart fitness platforms. The analysis plan anticipated positive associations throughout, and we retain those hypotheses rather than rewriting a boundary prediction after seeing the data. Following a reviewer’s advice, we state the hypotheses non-directionally and reserve the expected sign for the interpretation of the estimates: all tests reported here are two-sided, so the directional expectation never enters the test itself. Evidence for a separation between the two pathways would take the form of support for H1 and H2, non-support for H3 and H4, and convergent evidence that the cross-domain association is small enough to be of little practical consequence:

**H1a–c.**  *Perceived interaction is associated with subsequent social presence, flow, and belonging, controlling for their prior levels.*

**H2a–c.**  *Social presence, flow, and belonging are each associated with subsequent continuance intention when estimated simultaneously.*

**H3.**  *Continuance intention is associated with subsequent physical activity, controlling for prior physical activity.*

**H4a–c.**  *Perceived interaction has indirect associations with subsequent physical activity through each platform-domain experience and continuance intention.*

**RQ1**  Does physical activity prospectively predict perceived interaction in the reverse direction, which would threaten a one-directional reading of the platform chain?

The study makes three contributions. It converts a widely repeated continuity–behavior assumption into a four-wave prospective test in which the endpoint is the intention to continue rather than observed use, which is where the existing prospective evidence sits. It treats the asymmetry between a strong platform-domain chain and an absent cross-domain path as theoretically diagnostic, rather than presenting three familiar mediators as the contribution. And it bounds the cross-domain association with equivalence and Bayes-factor evidence, so that the absence of a crossing is reported as a bounded quantity rather than as a failed test.

## 2. Materials and Methods

### 2.1. Design and Procedure

We used a four-wave panel design with approximately four weeks between waves. Perceived interaction, social presence, flow, belonging, continuance intention, and physical activity were measured at T1 through T3; physical activity was measured again at T4, which was an outcome-only wave. Identical scoring rules were applied at every wave.

The interval was fixed in the protocol before data collection. Three considerations set it. The outcome instrument asks about exercise over the past month, so four-week spacing gives successive waves non-overlapping reference periods. The interval had to be long enough for perceived interaction to accumulate into the T2 experiences, yet short enough that the intervening period was not dominated by seasonal change in the opportunity to exercise. And four waves at this spacing kept the field period inside a single season, which matters for a physical activity outcome at this latitude.

Longitudinal linkage was pseudonymized. Participants generated a three-element study code from four digits representing birth month and day, the uppercase first letter of the mother’s surname in pinyin, and the uppercase pinyin initial of the first character of their primary school’s name. Contact details used for follow-up reminders and compensation were collected on a separate form, stored apart from survey responses, and scheduled for deletion within 30 days after T4 and completion of compensation. Analysis files use opaque random pseudonyms; the crosswalk between pseudonyms and study codes is held in a restricted file that is not part of the submitted data.

The study protocol was reviewed and approved by the Life Science Ethics Committee of Zhengzhou University on 2 December 2024, nine months before baseline data collection began. The approved protocol document specifies the four-wave design and its spacing, the planned fielding windows, the target sample of 550 with its age-stratum quotas, the a priori quality-screening rules, and the intended analysis. Questionnaire programming and pilot testing were completed in August 2025. T1 was administered from 14 to 27 September 2025 through the Wenjuanxing online survey platform (Changsha Ranxing Information Technology Co., Ltd., Changsha, China; https://www.wjx.cn, accessed on 12 September 2026) and its sample panel, using more than 46 quota-targeted panel cells. T2 ran from 12 to 25 October 2025, T3 from 9 to 22 November 2025, and T4, an outcome-only wave, from 7 to 20 December 2025. Each wave opened 28 days after the preceding wave, and the corresponding closing dates were likewise 28 days apart, so the achieved schedule matched the approximately four-week spacing specified in the protocol. Recruitment targets were 140 participants aged 18–25, 140 aged 26–35, 135 aged 36–45, and 135 aged 46–60; the achieved baseline file contained 130, 181, 130, and 109 records in those groups, so the 26–35 group was over-recruited and the 46–60 group under-recruited relative to target. No single gender exceeded 70% of the sample.

For participants aged 18–45, recruitment was nationwide, and the panel implemented within-age-group city-tier targets of approximately 15% first-tier, 25% new first-tier, 25% second-tier, and 35% third-tier or smaller; university and fitness-community channels were supplementary. Participants aged 46–60 were recruited primarily through community sports associations, square-dance groups, and universities for older adults in Zhengzhou using on-site QR-code access. We report the observed city-tier distribution rather than treating target shares as achieved.

Age was computed in completed years at the T1 survey date from reported month and year of birth, with a logic check; participants retained their T1 age group if they had a birthday between waves. Respondents aged 61 or older were screened out before quota enrollment. Eligibility further required the ability to complete a smartphone survey independently, use of a smart fitness platform, application, or wearable device within the previous three months, and access to a community physical activity venue or organization. Professional athletes, physical-education majors, and people medically advised against exercise were ineligible. Eligibility also required an affirmative response to a prescreening item, “During the past three months, have you used the community functions of a smart fitness platform, application, or wearable device (for example, browsing or posting updates, commenting or interacting with other members, or taking part in leaderboards)?” Every participant in the quality-screened analytic cohort had passed this prescreen. The item was used for eligibility only and was not retained in the de-identified analytic file, so the degree of community-function use is not available as a variable. Participants received a cash incentive of approximately CNY 50 for taking part, delivered through the survey platform.

### 2.2. Participants, Quality Screening, and Attrition

The baseline file contained 550 records. Before any analysis, a baseline record was excluded if completion time fell below one third of the T1 median, if the longest run of an identical response reached 15 items or more, or if either reverse-check discrepancy met the a priori threshold (|MR1+REV1−8|≥4 or |BI1+REV2−8|≥4; the constant 8 is the symmetric midpoint of the seven-point response scale); REV2, the reverse-check item paired with BI1, read “I plan to stop using the functions of this fitness community in the coming period.” These rules excluded 44 records, leaving 506. Screening preceded construction of the longitudinal cohort, and Table 1 reports the resulting sample flow.

At baseline, 269 participants (53.2%) were women and 237 (46.8%) were men. Education was high school or below for 129 (25.5%), associate degree for 143 (28.3%), bachelor’s degree for 177 (35.0%), and master’s degree or above for 57 (11.3%). Participants were located in first-tier (14.8%), new first-tier (19.2%), second-tier (29.8%), third- or fourth-tier (26.3%), and county-level or smaller (9.9%) areas. Mean platform and community use was 3.29 years (SD = 1.67). The screened cohort included 117 participants aged 18–25, 167 aged 26–35, 121 aged 36–45, and 101 aged 46–60.

### 2.3. Measures

Unless otherwise indicated, items used seven-point agreement scales. Composite scores were computed as the mean of available items within a scale and wave; a composite was formed only when a majority of that scale’s items were present, and the divisor was the number of observed items.

**Perceived interaction** was formed from three four-item dimensions—modality richness, bidirectionality, and response immediacy—adapted from work on media richness and interactivity (Daft & Lengel, 1986; Y. Liu, 2003; S. J. McMillan & Hwang, 2002). The index is the mean of the twelve items, which is equivalent to the mean of the three dimension scores because the dimensions contain equal numbers of items. It records what a participant perceives the platform to afford, not what the participant did on it; no behavioral usage log was collected in this study. Consistent with the formative logic set out in Section 1.2, the index is evaluated through indicator collinearity rather than internal consistency.

**Social presence** was measured with five items adapted from research on mediated presence (Gefen & Straub, 2003; Short et al., 1976). **Flow** used the nine-item Chinese short form (W. Liu, 2010) based on the short Flow State Scale (Jackson et al., 2008). **Belonging** used eight items based on sense-of-community research and its Chinese virtual-community validation (Blanchard & Markus, 2004; Cui & Chang, 2009; D. W. McMillan & Chavis, 1986). **Continuance intention** was measured with three items adapted from expectation-confirmation research (Bhattacherjee, 2001); the items refer to continued use of the fitness community, not to intention to exercise.

**Physical activity** was assessed with the Physical Activity Rating Scale-3 (PARS-3), translated and revised for Chinese use (Liang, 1994). Its three items assess exercise intensity, duration, and frequency, and the score is their product, with intensity and frequency coded 1–5 and duration coded 0–4, giving a range of 0 to 100. Scores of 0–19, 20–42, and 43–100 denote low, moderate, and high activity, and reported test–retest reliability is 0.82. In every wave of the present data, the stored total equals the product of the three items exactly. Because the score is a product and duration is coded from zero, the instrument has a true floor rather than a merely skewed lower tail: a respondent in the lowest duration category scores zero whatever their reported intensity and frequency. Zero scores number 8 at T1, 4 at T2, none at T3, and 24 at T4, and every zero in the data arises this way (Table S11). PARS-3 measures general physical activity and does not identify whether activity occurred offline, followed a livestream, or was attributable to the community; the title and conclusions therefore refer to subsequent physical activity rather than to online-to-offline conversion.

Age group, gender, education, city tier, and years of platform use were included as covariates in every equation, with identical coding in the primary specification, the complete-case checks, the imputation model, and the weighting model. Age group is an ordinal integer 0–3 for the strata 18–25, 26–35, 36–45, and 46–60; gender is an indicator with female coded as 1; education is an ordinal integer 1–4 for senior secondary or below, junior college, bachelor’s, and master’s or above; city tier is an ordinal integer 1–5 running from county level or below to first tier; and years of platform use is continuous (*M* = 3.29, *SD* = 1.67).

### 2.4. Analytic Strategy

The analysis plan, including the primary endpoint, the sampling targets, the quality screening rules, and the estimator, was specified a priori and is documented in the study protocol approved by the Life Science Ethics Committee of Zhengzhou University on 2 December 2024, nine months before baseline data collection. That approval provides an institutional date stamp on the plan. It is not a trial registration, and the study was not registered with a third-party registry.

Scale scores are the composites defined in Section 2.3. The archived data file also stores an integer-rounded copy of those composites, which does not follow that rule and is not used here; Section 3.6 re-estimates the focal equation from it.

The primary analysis estimated the joint multivariate-normal mean and covariance structure by full-information maximum likelihood, retaining all 506 screened participants under a missing-at-random assumption (Enders & Bandalos, 2001). Standardized path coefficients and equation $R^{2}$ values were derived from the FIML covariance matrix, and uncertainty was estimated with 2000 participant-level bootstrap resamples. The unified parallel–serial system estimated perceived interaction at T1 to social presence, flow, and belonging at T2, then to continuance intention at T3, then to physical activity at T4, in a single specification; each equation controlled the corresponding prior outcome where available plus the demographic covariates. Indirect effects were recomputed in every bootstrap resample so that cross-equation dependence was preserved (MacKinnon et al., 2004). All tests are two-sided.

Because H1, H2, and H4 each comprise three component tests, we applied Benjamini–Hochberg false discovery rate control within each a priori hypothesis family: H1a–c, H2a–c, the cross-domain family comprising H3 together with the three indirect effects, and the two exploratory reverse paths of RQ1, corrected separately and labeled exploratory. Adjusted *p* values appear in Table S5.

Sensitivity analyses used 20 stochastic chained-equation imputations pooled by Rubin’s rules with HC3 standard errors, complete cases, a log(1 + PARS-3) outcome, stabilized inverse-probability weights for T4 retention, and a standardized ordinary least squares recomputation as an implementation check. A person-mean-centered lagged model with participant-clustered standard errors examined within-person deviations; it is a sensitivity analysis and not a fully identified random-intercept cross-lagged panel model, and cross-lagged causal language is avoided accordingly (Hamaker et al., 2015).

Because the plan specified a positive H3 rather than a null, the equivalence tests and the Bayes-factor approximation reported in Section 3.5 are explicitly exploratory. Two one-sided tests were conducted at standardized bounds of ±0.05, ±0.10, and ±0.15, with all three reported so that no bound is selected for crossing a significance threshold (Lakens et al., 2018), together with a BIC approximation to the Bayes factor (Wagenmakers, 2007). A sensitivity power analysis was computed for the focal equation at α = 0.05 and power of 0.80.

Internal consistency, single-factor confirmatory models, and longitudinal configural, metric, and scalar models were fitted for each reflective item set and judged by the relative change in CFI (F. F. Chen, 2007; Cheung & Rensvold, 2002); the procedure is described in Supplementary Methods S1. Because absolute fit for the belonging item set was the weakest of the reflective sets, the structural analysis uses observed composites and does not claim latent mean change.

Analyses were conducted in Python 3.13.5 with NumPy 2.2.5, pandas 2.2.2, SciPy 1.15.3, statsmodels 0.14.6, and scikit-learn 1.6.1; multiple imputation used the scikit-learn iterative imputer with posterior sampling. The estimator, the confirmatory factor models, and the invariance tests are implemented directly in the accompanying code rather than through an external structural equation package, because semopy 2.3.11 returns an incorrect independence baseline under missing data and therefore reports negative comparative fit indices for well-fitting models; here the saturated model is estimated by expectation–maximization and the independence model in closed form. The pipeline regenerates every reported numerical result and all table inputs from the supplied data under a fixed seed, and accompanies the manuscript as Script S1; the published figures were drawn from those outputs. Runtime and package versions are recorded in run_log.json. Because rebuilding the pipeline also corrected the scoring rule described above, Table S12 reconciles every quantity reported in the previously submitted version against its rebuilt value.

### 2.5. Ethics

The study protocol was approved by the Life Science Ethics Committee of Zhengzhou University on 2 December 2024 (ZZUIRB 2024-27). All participants provided electronic informed consent before beginning the T1 questionnaire. The pseudonymization and contact-data separation procedures are described in Section 2.1.

## 3. Results

**Figure 2:**
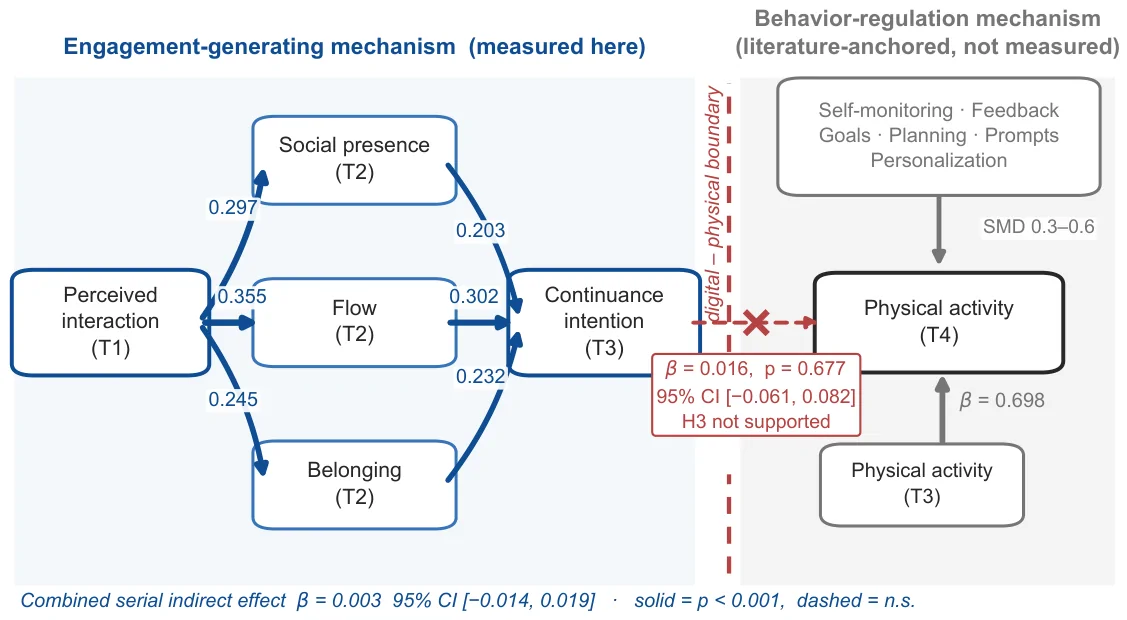
Estimated system: the platform chain holds; the crossing does not. Standardized coefficients from the unified FIML path system (*N* = 506 after quality screening; 2000 participant-level bootstrap resamples). Solid arrows denote *p* < 0.001 and the dashed red arrow denotes a non-significant path. Every equation controls for the corresponding prior outcome, age group, gender, education, city tier, and years of platform use. The autoregressive path from T3 to T4 physical activity (β = 0.698) is shown explicitly because it bounds the residual variance available to the focal predictor; Figure 3a shows that the cross-domain association is already near zero before any control is applied. The grey box is the same unmeasured organizing concept as in Figure 1.

**Figure 3:**
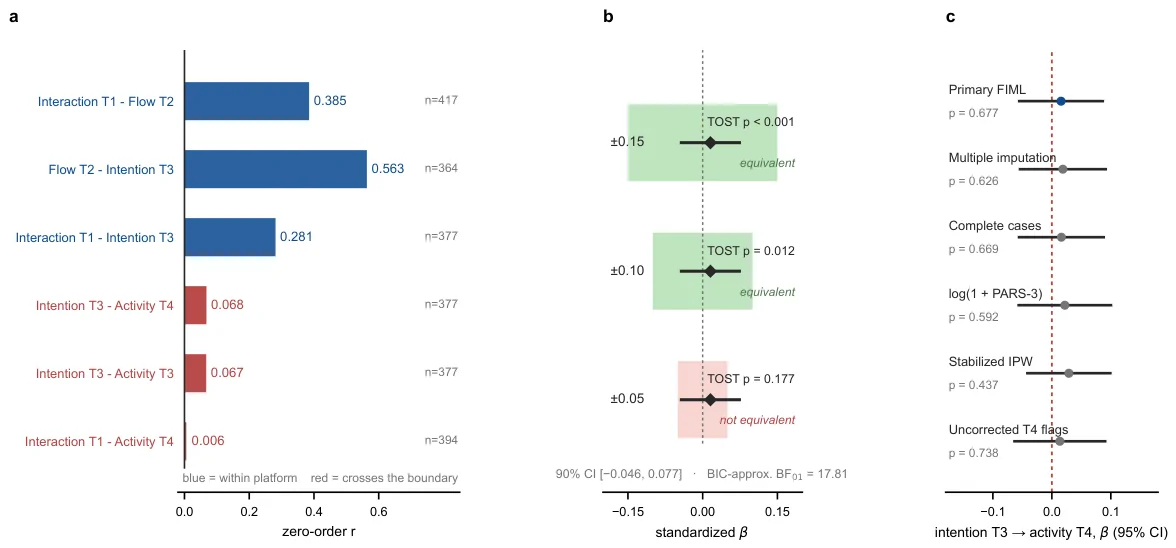
Where the measured engagement pathway stops. (**a**) Zero-order correlations on the quality-screened cohort before any covariate adjustment; blue denotes associations within the platform domain and red denotes associations that cross the digital–physical boundary. Pairwise *n* is given for each estimate. (**b**) Exploratory equivalence tests of the focal coefficient against three bounds for the smallest effect size of interest; the 90% confidence interval is [−0.046, 0.077] for all three comparisons, green shading marks bounds at which equivalence is established and red shading marks the bound at which it is not. (**c**) The focal coefficient across six estimation specifications, with 95% confidence intervals.

The argument of this section can be stated before the estimates. The chain that sustains the intention to keep using the platform holds at every link: perceived interaction predicts the three platform-domain experiences, and each of those predicts that intention. The chain then stops: that intention does not predict later physical activity, and the association was already near zero before any adjustment. The remainder of the section establishes how small the crossing can be said to be, and shows that the answer does not change under any of the specifications we could construct. Readers who do not follow the estimation detail can read Figure 2 and Figure 3, which carry the same three claims.

### 3.1. Preliminary Analyses

The supplied baseline file contained 550 records. Applying the a priori quality rules excluded 44 records, leaving an analysis cohort of 506. Recalculation from the item-level T1 file reproduced the stored quality flag exactly: 19 records were faster than one third of the T1 median completion time of 290 s, 27 had a longest identical-response run of at least 15 items, and two records met more than one rule. The two reverse-check rules identified no additional cases. Both reverse-check items showed almost no variance in this sample (observed values 3 and 4 only), so those rules had little practical sensitivity here; the exclusions were driven by completion time and long-string responding. Screening was completed before the longitudinal cohort was assembled.

Retention was 417 at T2 (82.4%), 377 at T3 (74.5%), and 394 at T4 (77.9%), with the quality-screened T1 cohort as the denominator throughout (Table 1). T4 retention exceeds T3 retention because T4 was an outcome-only wave whose completion was defined from the outcome itself: all three PARS-3 items had to be observed, and the stored total had to equal their product. During the data audit, 40 source records met this rule while carrying an outdated retention flag; 38 of them belonged to the quality-screened cohort, raising T4 retention from 356 to 394. A record-level correction log accompanies the reproducibility materials as Table S1.

All 38 records show the same pattern: a complete T4 item set whose stored total equals the product of its three items, retention at T3, and session-level fields that are jointly absent or negative. Because the flag is derived from the session record rather than from the item responses, these are records with T4 item responses and no T4 session record. The source export retained an outdated T4 status flag for these records. The archived materials do not preserve the platform-side logs that would establish why the corresponding session record was never written, so we describe the discrepancy that the record-level audit can establish rather than assign an operational cause we cannot verify. Recomputing T2 and T3 retention by the same logic returns 417 and 377 with no mismatch against the stored flags, so the discrepancy is confined to T4. The reclassified records do not differ from the 356 already flagged on the outcome (*M* = 20.13, *SD* = 17.59 versus *M* = 20.02, *SD* = 15.93), and Section 3.6 reports the focal estimate under the uncorrected flags. Supplementary Methods S2 gives the full audit.

A multivariable logistic attrition model predicted retention at each wave from baseline constructs and demographics (Table S8). Baseline physical activity was not detectably associated with T4 retention (OR = 0.964, 95% CI [0.770, 1.207], *p* = 0.750), whereas baseline continuance intention was (OR = 2.968, 95% CI [2.241, 3.932], *p* < 0.001). Because 22.1% of the screened cohort did not complete T4, missing-data and weighting analyses are reported alongside the primary specification, and Section 3.5 examines directly whether selective retention on the focal predictor restricts its range.

Measurement was evaluated before any structural estimates. Across the reflective scales, Cronbach’s α and McDonald’s ω both ranged from 0.919 to 0.976 (Table 2); single-factor confirmatory models at T1 returned standardized loadings between 0.892 and 0.941 (Table S2); and longitudinal configural, metric, and scalar models converged for every repeated construct, with configural CFI from 0.989 to 1.000, RMSEA from 0.000 to 0.045, and no constraint changing CFI by more than 0.0013 (Table S3). The scores are therefore comparable across waves.

Perceived interaction is formative and is instead evaluated through the association among its dimensions. Variance inflation factors ranged from 1.000 to 1.006 and the raw pairwise correlations from −0.059 to 0.073 (Table S4), so the three dimensions are close to statistically independent in this sample. That is permitted by a formative composite rather than required by a reflective one, but it warrants a direct test, and Section 3.6 reports the model with the three dimensions entered separately. We return to what the index represents in Section 4.3.

### 3.2. Descriptive Statistics and Zero-Order Associations

Table 3 reports means, standard deviations, and zero-order correlations for the focal variables before any covariate adjustment. Two patterns are visible, and their contrast carries most of the paper’s argument.

Within the platform domain, associations are of ordinary magnitude: perceived interaction at T1 correlated 0.385 with flow at T2 and 0.281 with continuance intention at T3, and flow at T2 correlated 0.563 with continuance intention at T3; across the six pairs adjacent along the hypothesized chain, the correlations run from 0.218 to 0.563. Every association that crosses the digital–physical boundary is close to zero: continuance intention at T3 with physical activity at T4, *r* = 0.068 (*n* = 377); continuance intention and physical activity measured at the same wave, *r* = 0.067 (*n* = 377); perceived interaction at T1 with physical activity at T4, *r* = 0.006 (*n* = 394). Physical activity was strongly autocorrelated (T3 with T4, *r* = 0.691).

This matters for how the focal test in Section 3.4 should be read. Because the autoregressive path is strong, one could expect any additional predictor of T4 activity to have little residual variance left to explain. The zero-order results show that this is not the operative reason: the cross-domain association is already indistinguishable from zero before prior activity or any covariate is entered. Figure 3a displays this contrast.

### 3.3. The Platform-Domain Chain

Controlling prior levels and demographics, perceived interaction at T1 predicted all three platform-domain experiences at T2: social presence (β = 0.297, 95% CI [0.220, 0.371]), flow (β = 0.355, 95% CI [0.285, 0.422]), and belonging (β = 0.245, 95% CI [0.175, 0.318]). H1a–c were supported. Entered simultaneously, each T2 experience predicted continuance intention at T3: social presence β = 0.203, flow β = 0.302, and belonging β = 0.232 (all *p* < 0.001). H2a–c were supported, and all six paths remain significant at *p* < 0.001 after Benjamini–Hochberg correction within their families (Table S5). Table 4 reports the full system and Figure 2 displays it.

An independent recomputation using standardized ordinary least squares on complete cases returned 0.294, 0.357, and 0.243 for the three H1 paths and 0.203, 0.298, and 0.221 for the three H2 paths, so the results do not depend on the FIML implementation. The two estimators are computed from identical composite scores; the small residual differences reflect the treatment of missing data rather than a difference in specification.

### 3.4. The Digital–Physical Boundary

Continuance intention at T3 was not detectably associated with physical activity at T4 once T3 activity and covariates were controlled (β = 0.0155, bootstrap SE = 0.0373, 95% CI [−0.061, 0.082], *p* = 0.677). Neither perceived interaction at T3 (β = 0.0118, 95% CI [−0.065, 0.087], *p* = 0.763) nor perceived interaction at T1 (β = 0.0175, 95% CI [−0.060, 0.096], *p* = 0.665) was associated with T4 activity in the same equation. Prior activity was the dominant predictor (β = 0.698, 95% CI [0.623, 0.761]). H3 was not supported. The complete-case recomputation agreed (β = 0.0162, *p* = 0.669 for continuance intention). Because PARS-3 records exercise wherever it occurred, this null cannot be produced by an outcome measure that was blind to activity taking place away from the platform. The four cross-domain tests remain non-significant after Benjamini–Hochberg correction within their family (adjusted *p* = 0.687; Table S5).

Because the final path was near zero, none of the three serial indirect effects were distinguishable from zero. The combined indirect effect was β = 0.0035, 95% CI [−0.0138, 0.0190] across 2000 bootstrap resamples, and the 20-imputation analysis agreed (combined β = 0.0039, 95% CI [−0.0014, 0.0109]). H4a–c were not supported (Table 5). Following the recommendation to report the constituent paths rather than the product alone, we note that the a-paths (interaction to each experience) and the b-paths (each experience to intention) were all supported; what fails is the final cross-domain segment, not the mediation chain within the platform.

### 3.5. How Small Is the Cross-Domain Association?

A non-significant coefficient does not by itself establish the absence of an effect, so we quantified how small the association can be said to be. The analysis plan specified a positive H3; it did not specify a null or an equivalence hypothesis. The equivalence bounds below were therefore chosen after the focal coefficient was found non-significant and are reported as an exploratory sensitivity range rather than as a confirmatory test, with all three bounds reported so that no single interval is selected for crossing a threshold (Lakens et al., 2018).

The 90% confidence interval for the focal coefficient was [−0.046, 0.077]. Two one-sided tests rendered effects at least as large as ±0.15 (*p* = 0.0002) and ±0.10 (*p* = 0.012) implausible, but not ±0.05 (*p* = 0.177) (Table S6, Figure 3b). Rather than read these as a binary verdict, we report alongside each bound the power the test would have had if the true effect were exactly zero: 0.00 at ±0.05—that bound is narrower than 1.645 bootstrap standard errors, so equivalence there was unattainable by construction—0.70 at ±0.10, and 0.98 at ±0.15. The outcome at ±0.05 therefore reflects the resolution of the study rather than evidence of an effect of that size. A BIC approximation to the Bayes factor (Wagenmakers, 2007) favored the model without the focal path, BF_01_ = 17.81. Because a standardized coefficient of 0.10 is roughly 1.61 PARS-3 points per standard deviation of continuance intention here, the bounded region renders associations conventionally described as small or larger implausible (Gignac & Szodorai, 2016) while remaining compatible with very small ones. With *n* = 377, α = 0.05, and a power of 0.80, the smallest detectable standardized coefficient is 0.104, which agrees with the observed bootstrap standard error of 0.0373.

A distinct threat is that selective retention restricted the range of the focal predictor, since baseline continuance intention predicted staying in the study. The comparison must be made at T1, because continuance intention at T3 is observed only for T3 completers and every T3 completer also completed T4 (377 of 377). Split by T3 retention, baseline continuance intention had *M* = 5.054, SD = 0.958 among those retained and *M* = 4.212, SD = 0.738 among those not (Levene’s test on the T4 split, *p* = 0.001; Table S10). Dropouts were therefore both lower and less variable. The consequence is small: because they are a minority, the retained subsample preserves 98% of the full cohort’s dispersion (SD 0.958 against 0.978), an attenuation factor of 1.021 that moves the focal coefficient from 0.0155 to 0.0158—far short of the ±0.10 bound. The weighted specification in Section 3.6, which models retention explicitly, likewise returns a null.

### 3.6. Robustness and Exploratory Analyses

Across six specifications, the focal coefficient stayed between 0.005 and 0.029 and was never detectable (Table S7, Figure 3c): 20 multiple imputations, complete cases, a log-transformed outcome, stabilized inverse-probability weighting for T4 retention, the uncorrected retention flags, and the integer-rounded composites in the archived file. Two of them bear on objections raised elsewhere in this paper. The uncorrected flags retain 356 rather than 394 participants and leave the estimate unchanged (β = 0.0135, *p* = 0.738, *n* = 339), so the flag reconciliation does not drive the result; the rounded composites return β = 0.0051 (*p* = 0.887), so neither does the scoring rule. Because the outcome has a floor as well as a right skew, the log transformation is a check on skew rather than on the floor.

Three exploratory analyses qualify the platform-domain findings. Neither reverse path from physical activity to perceived interaction was detectable (T1 to T2, β = −0.0050, *p* = 0.881; T2 to T3, β = 0.0669, *p* = 0.102), so these data do not support a reciprocal-feedback account. In person-mean-centered lagged models with participant-clustered standard errors, a higher-than-usual perceived-interaction score predicted higher subsequent flow (β = 0.175, *p* < 0.001) and social presence (β = 0.130, *p* = 0.020), with belonging short of conventional significance (β = 0.089, *p* = 0.063); two of the three links therefore hold when only within-person deviations are used. Finally, entering the three interaction dimensions separately in place of the index leaves the pattern intact: each independently predicted each T2 experience (β from 0.133 to 0.235, all *p* < 0.001) with an essentially unchanged equation $R^{2}$, and none predicted T4 activity (Table S9). The cross-domain null does not depend on aggregating the dimensions.

## 4. Discussion

This four-wave study produced an asymmetry. Perceived interaction prospectively predicted social presence, flow, and belonging, and each of the three independently predicted continuance intention. The chain that sustains the intention to keep using the platform is intact and replicates across estimators. It then stops at the platform boundary: continuance intention did not predict subsequent physical activity once prior activity was controlled, and the serial indirect effects were indistinguishable from zero. The result held across six specifications spanning missing-data treatment, outcome transformation, selective retention, the record-level flag correction, and the scoring of the composite scores.

### 4.1. The Null Is Not an Artifact of Conditioning

The most direct objection to a null of this kind is that it was manufactured by the model. Prior activity predicted subsequent activity strongly (β = 0.698), and once such an autoregressive path is included, little residual variance remains for any other predictor.

The zero-order results answer this objection. Before prior activity or any covariate enters the equation, continuance intention and subsequent physical activity correlate at 0.068; measured at the same wave, they correlate at 0.067; and perceived interaction and later activity correlate at 0.006. Associations along the hypothesized within-platform chain, over the same waves and the same participants, run from 0.218 to 0.563. The crossing is absent in the raw associations, not removed by conditioning. Nor is the null what an insensitive-instrument account would predict: the same instrument tracked itself from T3 to T4 at *r* = 0.691, so the null is not attributable to an outcome measure that fails to register activity. Reporting both layers is what allows the null to be interpreted rather than merely reported.

### 4.2. Why the Chain Stops Where It Does

Three accounts are consistent with the pattern. The first two are complementary rather than competing; the third is a threat to inference that the data can bound but not eliminate.

The first concerns what engagement experiences supply. Social presence, flow, and belonging give a person reasons to return to a community. They do not supply plans, action triggers, scheduled occasions, or performance feedback. The evidence base for digital tools that do move activity is built on interventions defined by automated self-monitoring and feedback (Ferguson et al., 2022; Laranjo et al., 2021). Our data locate the boundary of the other mechanism; they do not test the scaffolding one, because none of its components were manipulated or measured here.

The second concerns the outcome. Intentions lose predictive power over behaviors that have become habitual and context-bound (Ajzen, 2002; Ouellette & Wood, 1998), and the same limit is documented for continued technology use (S. S. Kim et al., 2005; Limayem et al., 2007). Participants in this study were established users with access to an activity venue, and physical activity behaved accordingly: it was the most stable variable in the system, with an autoregressive coefficient of 0.698. A platform-directed intention has little purchase on a behavior that is already running on habit. On this reading, the finding is not an absence of a relationship that ought to exist, but the expected consequence of asking a deliberative, domain-external variable to move an established routine.

The third account is methodological rather than substantive, and it is a genuine alternative to the habit reading rather than a variant of it. Because baseline continuance intention predicted staying in the study, the participants who supply the T3–T4 estimate are drawn disproportionately from the upper part of its distribution, and a restricted predictor attenuates the coefficient estimated from it. The quantification in Section 3.5 bounds this at about 2% attenuation, which is not enough to overturn the conclusion; the bound is what the data support, not a demonstration that no restriction occurred.

This also sharpens what would count as evidence against a separation between the two pathways. If the crossing appeared once intention were coupled to scheduled sessions or verifiable action prompts, or among users who have not yet established an exercise routine, the boundary would be conditional rather than structural. Both are testable.

### 4.3. Levels of Inference and the Structure of the Interaction Index

Two results qualify how the platform-domain chain should be read.

First, the conventional lagged models supported all three routes, and the person-mean-centered analyses supported two of them, with belonging falling just short. Stable between-person differences may therefore account for part of the belonging pattern, so the chain is best described as a set of prospective between-person associations with within-person support established for flow and social presence.

Second, the three interaction dimensions are close to statistically independent in this sample: variance inflation factors between 1.000 and 1.006 and raw pairwise correlations between −0.059 and 0.073. Low collinearity is a condition a formative composite permits, but it is not on its own a reason to combine three facets into one equally weighted index, and we no longer present it as one. The reason for retaining the index is empirical: entering the three dimensions separately reproduces the same conclusions with essentially the same explanatory power (Table S9), so the aggregation is lossless here. What the near independence does establish is what the index represents. Perceived interaction is a record of what the platform provides—three design provisions that can be varied one at a time—and not a unitary psychological construct that the three facets jointly indicate. It should be read that way, and a reader who prefers the disaggregated model can read it instead.

### 4.4. Theoretical Implications

The study separates platform-domain continuity from cross-domain behavior, which keeps continuance intention from being read as an exercise intention and turns an implicit assumption into a direct prospective test. The a priori positive H3 was not supported, and we do not retroactively redefine it as a null prediction.

It also reframes the contrast in the literature as engagement-generating versus behavior-regulation mechanisms. The present data show that interaction, social presence, flow, and belonging form a coherent engagement pathway; the trial and review evidence shows that digital tools change activity when they deliver self-monitoring, feedback, goals, and prompts. Because this study measured only the first mechanism, the contrast is an evidence-grounded interpretation rather than a head-to-head comparison.

Finally, it formalizes digital-to-physical spillover as an outcome distinct from digital continuity, and it reports the absence of that spillover as a bounded quantity rather than as a failed test. Exploratory equivalence testing rendered standardized associations of ±0.10 or larger implausible, with a power of 0.70 at that bound had the true effect been zero, and the Bayes-factor approximation favored the model without the path. Neither reverse path was detectable, so we do not infer a reciprocal loop.

### 4.5. Practical Implications

Platform continuity and physical activity should be evaluated as separate outcomes. Rich media, reciprocal exchange, and rapid response were prospectively associated with social presence, flow, and belonging and, through them, with the intention to keep using the community; sustaining that intention is a legitimate design goal on its own terms. It is not evidence of a health effect, and an engagement endpoint—here the intention to continue and, by extension, the usage metrics taken to stand in for it—should not be reported as though it were one.

The more useful reading is that continued engagement is at most a precondition, and one that has to be spent on something. A community that sustains the intention to return but supplies no action regulation has, based on the present evidence, no further claim on behavior. Because belonging was the experience most anchored in durable social ties, the natural test is whether the relationships that underlie belonging can be made to carry accountability structures—committed training partners, scheduled group sessions, and verified completion—rather than adding generic prompts unconnected to the community. A factorial design that crosses engagement features with action-regulation features, while measuring both platform use and device-recorded activity, would show whether engagement improves adherence to an effective scaffold, whether scaffolds work without social immersion, or whether the two interact.

### 4.6. Cautions for Commissioning, Implementation, and Evaluation

The separation documented here bears on how digital physical activity services are specified and evaluated, because engagement—retention in practice, and the intention to continue in the present data—is increasingly treated as an accountability metric in publicly funded digital health. What follows is put as cautions, and as questions for a future evaluation to answer, rather than as recommendations this design can license: the bounds set out in Section 4.7—a self-reported outcome, no behavioral usage log, and a sample of established users—apply to every point in it.

For commissioning, these data give one reason to be careful with engagement endpoints. The intention to keep using a platform and subsequent behavior were separable here, so a service could meet an engagement target in full while producing no detectable change in the behavior the programme exists to change. Whether observed retention behaves as the intention endpoint did is untested here, and worth establishing before either is written into a specification as a proxy for activity.

For implementation, the case for treating engagement features and action-regulation features as distinct components with distinct acceptance criteria rests on the two pieces of literature used, at least as much as on these data. The components that generate social presence, flow, and belonging were reproducible in this sample; nothing measured here shows that they substitute for the self-monitoring, goal-setting, feedback, and prompting components on which the intervention evidence base rests (Ferguson et al., 2022; Laranjo et al., 2021).

For evaluation, the point we would defend is methodological rather than programmatic. An evaluation that carries a usage endpoint should carry a behavioral endpoint alongside it, state in advance the smallest behavioral effect that would justify continued investment, and report interval-based conclusions when that effect is not observed. A null reported as a bounded quantity supports a decision; a null reported only as a non-significant coefficient does not.

### 4.7. Limitations

Three bounds define the scope of these conclusions. The outcome is self-reported general physical activity measured by PARS-3, with a floor at zero and no attribution of activity to its source, so the findings are specific to that measure; device-based measurement and feature-level usage logs would be required to generalize beyond it. The predictor is the intention to continue rather than observed use, and no behavioral usage log was collected, so the study bounds what the intention endpoint carries and not what continued use carries. Eligibility required current platform use, use of community functions, and access to an activity venue, but the prescreen was binary and did not measure how often or how intensively those functions were used, so the findings describe established users and do not extend to people who have not yet adopted a platform, for whom the engagement pathway may have more room to operate.

The analysis plan was specified a priori and is documented in the approved study protocol, but it was not registered with a third party, and the equivalence bounds were necessarily chosen after the focal coefficient was estimated. A confirmatory replication should fix the focal null, the smallest effect size of interest, and an objective outcome in advance.

## 5. Conclusions

Perceived interaction in online fitness communities supported social presence, flow, and belonging, and each of these supported the intention to keep using the community. That chain did not extend to behavior: continuance intention showed no prospective association with self-reported physical activity once prior activity was controlled, and the association was already near zero before any adjustment. Exploratory equivalence testing rendered cross-domain associations of ±0.10 or larger implausible while leaving very small associations inside ±0.05 possible. These are prospective associations in a longitudinal observational design, not an identification of distinct causal mechanisms, and they are specific to general physical activity as measured by PARS-3 in Chinese adults who were already using a smart fitness platform.

The implication is specific. Engagement design sustains the intention to stay involved, and the experiences that achieve this are measurable and reproducible. They should not be assumed to act as behavioral scaffolding, and an engagement endpoint should not be reported as a proxy for a health outcome. Where activity is the goal, the design question is what the sustained engagement is then used to deliver. 

## Figures and Tables

**Table 1:** Sample flow. Percentages use the quality-screened T1 cohort as the denominator. T4 exceeds T3 because T4 completion was defined from the outcome itself (all three PARS-3 items observed and the stored total equal to their product). Before the record-level correction described in Section 3.1, the source retention flag recorded 356 participants at T4 (70.4%); 38 records whose T4 item set was complete but whose session-level fields were absent were reclassified, giving 394. The correction log is Table S1, and Section 3.6 reports the focal estimate under the uncorrected flags.

Stage	*n*	% of Screened T1
Raw baseline records	550	—
Quality-screened T1 cohort	506	100.0
T2 retained	417	82.4
T3 retained	377	74.5
T4 retained (source flags)	356	70.4
T4 retained (after correction)	394	77.9

**Table 2:** Internal consistency across T1–T3 for the reflective scales. *N* varies because reliability was computed from complete items within each scale and wave. Perceived interaction is a formative index and is therefore assessed by indicator collinearity and by the association among its dimensions (Table S4) rather than by α or ω.

Construct	Items	α Range	ω Range	Min *N*	Max *N*
Social presence	5	0.919–0.964	0.919–0.964	377	506
Flow	9	0.962–0.976	0.962–0.976	377	506
Belonging	8	0.956–0.974	0.956–0.974	377	506
Continuance intention	3	0.927–0.939	0.927–0.939	377	506

**Table 3:** Descriptive statistics and zero-order correlations among focal variables, computed on the quality-screened cohort with pairwise available data and before any covariate adjustment. Correlations that cross the digital–physical boundary are shown in bold. Coefficients of |r|≥0.11 are significant at *p* < 0.05 at the smallest pairwise *n* in the table.

Variable	*n*	*M*	SD	1	2	3	4	5	6	7
1. Interaction T1	506	4.82	0.54							
2. Social presence T2	417	4.79	0.76	0.282						
3. Flow T2	417	4.81	0.77	0.385	0.286					
4. Belonging T2	417	4.81	0.78	0.218	0.013	0.127				
5. Intention T3	377	4.88	0.94	0.281	0.450	0.563	0.425			
6. Activity T1	506	19.49	14.93	**0.018**	**0.008**	**0.012**	**0.052**	**−0.014**		
7. Activity T3	377	19.58	11.37	**−0.016**	**−0.057**	**−0.069**	**0.133**	**0.067**	0.333	
8. Activity T4	394	20.03	16.08	**0.006**	**−0.063**	**−0.067**	**0.139**	**0.068**	0.182	0.691

**Table 4:** Unified FIML path system. *N* = 506 after quality screening, with 2000 participant-level bootstrap resamples. All equations include the prior outcome where available and the demographic covariates. Coefficients are standardized; *p* values use the bootstrap standard-error normal approximation and intervals are percentile bootstrap intervals. Adjusted *p* values under Benjamini–Hochberg control within hypothesis families are in Table S5.

Path	β	SE	95% CI	*p*	$R^{2}$
Interaction T1 → presence T2	+0.297	0.037	[+0.220, +0.371]	<0.001	0.534
Interaction T1 → flow T2	+0.355	0.034	[+0.285, +0.422]	<0.001	0.610
Interaction T1 → belonging T2	+0.245	0.036	[+0.175, +0.318]	<0.001	0.490
Presence T2 → intention T3	+0.203	0.034	[+0.133, +0.268]	<0.001	0.703
Flow T2 → intention T3	+0.302	0.031	[+0.238, +0.360]	<0.001	0.703
Belonging T2 → intention T3	+0.232	0.033	[+0.166, +0.296]	<0.001	0.703
Activity T3 → activity T4	+0.698	0.036	[+0.623, +0.761]	<0.001	0.492
Intention T3 → activity T4	+0.016	0.037	[−0.061, +0.082]	0.677	0.492
Interaction T3 → activity T4	+0.012	0.039	[−0.065, +0.087]	0.763	0.492
Interaction T1 → activity T4	+0.017	0.040	[−0.060, +0.096]	0.665	0.492
Activity T1 → interaction T2	−0.0050	0.033	[−0.071, +0.060]	0.881	0.378
Activity T2 → interaction T3	+0.067	0.041	[−0.013, +0.146]	0.102	0.317

**Table 5:** Serial indirect effects from perceived interaction at T1 to physical activity at T4. FIML uses all 506 screened participants with 2000 bootstrap resamples; the missing-data sensitivity uses 20 chained-equation imputations. Coefficients are reported to four decimal places because all four are smaller in absolute value than 0.005.

Route	FIML Effect	FIML 95% CI	MI Effect	MI 95% CI
Via social presence	0.0009	[−0.0038, 0.0054]	0.0010	[−0.0004, 0.0026]
Via flow	0.0017	[−0.0067, 0.0095]	0.0019	[−0.0006, 0.0057]
Via belonging	0.0009	[−0.0037, 0.0050]	0.0009	[−0.0004, 0.0026]
Combined	0.0035	[−0.0138, 0.0190]	0.0039	[−0.0014, 0.0109]

## Data Availability

The pseudonymized participant-level dataset (Data S1), the complete primary analysis pipeline (Script S1), the verification script and its output (Script S2), and the record-level correction log (Table S1) are provided as Supplementary Materials with this article, and will also be deposited in a public repository upon publication. The crosswalk linking pseudonyms to participant-generated study codes is not shared, in accordance with the approved protocol.

## Supplementary Materials

The following supporting information can be downloaded at: https://www.mdpi.com/article/10.3390/bs16091645/s1, Table S1: The record-level T4 retention-flag correction log; Table S2: Standardized loadings from single-factor confirmatory models at T1; Table S3: Longitudinal measurement invariance; Table S4: Pairwise correlations and variance inflation factors among the perceived-interaction dimensions; Table S5: Benjamini–Hochberg-adjusted *p* values by hypothesis family; Table S6: Equivalence tests; Table S7: The focal coefficient across specifications; Table S8: Attrition models; Table S9: The model with the three interaction dimensions entered separately; Table S10: The focal predictor by retention status; Table S11: The PARS-3 distribution and its floor; Table S12: A reconciliation of every quantity reported in the previous version against the rebuilt pipeline; Data S1: The pseudonymized participant-level dataset; Script S1: The complete primary analysis pipeline, which regenerates every reported numerical result and all table inputs from Data S1 and from whose outputs the published figures were drawn; Script S2: The standard-library verification script distributed with the previous version, retained so that the independent recomputation it performs remains available.
