# Supplementary material for: From Digital Engagement to Physical Activity: A Four-Wave Test of Behavioral Spillover in Online Fitness Communities
Source: Behav Sci (Basel). 2026 Sep 14;16(9):1645. doi: 10.3390/bs16091645 (PMC13603695; doi:10.3390/bs16091645)
Supplement: Supplementary file 1 [file behavsci-16-01645-s001.zip › SI.pdf]

Supplementary Materials

From Digital Engagement to Physical Activity? A Four-Wave Test of Behavioral Spillover in Online Fitness Communities

This document accompanies manuscript behavsci-4505913. Every table is regenerated from the supplied data by Script S1; no value is transcribed by hand. Tables S2–S12 are new in this revision and were added in response to the first round of review.

Supplementary Methods

S1. Measurement models

Reliability was computed as Cronbach's α on complete item sets within each scale and wave, and as McDonald's ω from the standardized loadings of a single-factor confirmatory model fitted to the same items under full-information maximum likelihood.

Longitudinal invariance was assessed for each reflective item set with a three-wave single-factor model in which the residual of each item was allowed to correlate with the residual of the same item at the other two waves. The first item of each wave was fixed for identification. The configural model leaves loadings and intercepts free across waves with factor means fixed at zero; the metric model constrains loadings to equality across waves; the scalar model additionally constrains intercepts, freeing the factor means at T2 and T3. Models were compared by the relative change in the comparative fit index (Cheung and Rensvold, 2002; Chen, 2007).

Fit statistics were formed against a saturated model estimated by expectation–maximization on the observed data and an independence model estimated in closed form, both under the same missing-data likelihood as the target model. This is the reason the analysis does not use an external structural equation package: semopy 2.3.11 fits these models under full-information maximum likelihood but computes the independence baseline incorrectly when data are missing, returning negative comparative fit indices for models that fit well. The implementation is in `P03_measurement.py` of Script S1.

S2. Audit of the wave-level retention indicators

The T4 retention indicator supplied in the source export was reconciled against the item data before analysis. Completion at T4 was defined a priori from the outcome itself: all three PARS-3 items observed, and the stored total equal to their product. Applying that rule to the source file identified 40 records that satisfied it while carrying a negative retention flag, of which 38 belong to the quality-screened cohort. Table S1 lists them.

All 38 share one pattern. Each satisfies the completion rule and each was retained at T3, while every session-level field in the source export is jointly absent or negative: the T4 completion duration is missing for all 38 of 38, the last-wave marker records wave 3, and the retention flag itself is false. Because the flag is derived from the session record rather than from the item responses, these are records for which T4 item responses exist and no T4 session record does.

Because a stale indicator at one wave raises the question of whether the others are affected, retention at T2 and at T3 was recomputed from item-level completeness by the same logic. The recomputation returns 417 at T2 and 377 at T3 and agrees with the stored flags for every record at both waves. The discrepancy is confined to T4.

Two checks bear on whether the correction is selective. The rule was applied to all records meeting the outcome-completeness criterion irrespective of their values on any predictor. The outcome distribution of the 38 reclassified records is indistinguishable from that of the 356 already flagged ($M = 20.13$, $SD = 17.59$ versus $M = 20.02$, $SD = 15.93$), and the focal estimate under the uncorrected flags is reported in Table S7. The audit is executed by `run_all.py` of Script S1 and its output is recorded in `run_log.json`.

Supplementary Tables

Table S1: Record-level log of the T4 retention-flag correction. Each record listed carried a negative source retention flag while its T4 PARS-3 item set was complete and the stored total equalled the product of its three items. The log contains 40 source records, of which 38 belong to the quality-screened cohort; it is also supplied in full as a CSV file. The first ten rows are shown here.

pseudonym	in_quality_screened_cohort	stay_T4_source	stay_T4_corrected
P-7EX67PMB1J	yes	False	1
P-529VDQ4EDB	yes	False	1
P-MDS2ZYREJQ	yes	False	1
P-MTNDQJK5H3	yes	False	1
P-Y73EJA0THG	yes	False	1
P-DZR29ZFVKK	yes	False	1
P-SSNXN12XD1	yes	False	1
P-7JWV6J5PD1	yes	False	1
P-413C5VVWW2	yes	False	1
P-SXC8268P3A	yes	False	1

Table S2: Standardized loadings from single-factor confirmatory models fitted to each reflective scale at T1 under full-information maximum likelihood. Loadings are listed in item order.

Construct	Items	Standardized loadings	Range
Social presence	5	0.941 0.913 0.922 0.910 0.907	0.907–0.941
Flow	9	0.913 0.903 0.901 0.909 0.892 0.907 0.911 0.918 0.903	0.892–0.918
Belonging	8	0.909 0.916 0.905 0.907 0.907 0.907 0.909 0.911	0.905–0.916
Continuance intention	3	0.926 0.922 0.897	0.897–0.926

Table S3: Longitudinal measurement invariance across T1–T3 for each reflective item set. Models were fitted under full-information maximum likelihood with the first item of each wave fixed for identification and residuals of the same item correlated across waves. The chi-square is referenced to the saturated model estimated by expectation–maximization and the comparative fit index to the independence model in closed form.

Construct	Level	χ^2	df	CFI	TLI	RMSEA	ΔCFI
Social presence	Configural	144.88	72	0.9885	0.9833	0.0448	—
Social presence	Metric	150.83	80	0.9889	0.9854	0.0419	+0.0003
Social presence	Scalar	154.25	88	0.9896	0.9876	0.0386	+0.0007
Flow	Configural	397.51	294	0.9924	0.9909	0.0264	—
Flow	Metric	407.76	310	0.9928	0.9919	0.0250	+0.0004
Flow	Scalar	419.16	326	0.9932	0.9926	0.0238	+0.0003
Belonging	Configural	354.09	225	0.9892	0.9868	0.0337	—
Belonging	Metric	370.07	239	0.9890	0.9873	0.0330	−0.0002
Belonging	Scalar	384.56	253	0.9890	0.9880	0.0321	¡ 0.001
Continuance intention	Configural	9.96	15	1.0000	1.0033	0.0000	—
Continuance intention	Metric	23.63	19	0.9987	0.9976	0.0220	−0.0013
Continuance intention	Scalar	24.27	23	0.9997	0.9995	0.0105	+0.0009

Table S4: Diagnostics for the formative perceived-interaction index: variance inflation factors for the three dimensions (modality richness, bidirectionality, response immediacy) and the raw pairwise correlations among them, at each wave. Low collinearity is a condition a formative composite permits; it is not on its own a reason to aggregate, and Table S9 reports the model with the three dimensions entered separately.

Wave	VIF (MR / BI / RI)	MR–BI	MR–RI	BI–RI	n
T1	1.000 / 1.004 / 1.004	−0.011	−0.009	−0.059	506
T2	1.000 / 1.005 / 1.006	+0.004	−0.016	+0.073	417
T3	1.003 / 1.003 / 1.006	+0.005	−0.058	−0.050	377

Table S5: Benjamini–Hochberg false discovery rate control within each a priori hypothesis family. No decision changes under correction: the six platform-domain paths remain significant and the four cross-domain tests remain non-significant.

**Family**	**Test**	p	p_{BH}	**Sig. at .05**
H1 (interaction T1 → experiences T2)	Interaction T1 → social presence T2	¡0.001	¡0.001	Yes
H1 (interaction T1 → experiences T2)	Interaction T1 → flow T2	¡0.001	¡0.001	Yes
H1 (interaction T1 → experiences T2)	Interaction T1 → belonging T2	¡0.001	¡0.001	Yes
H2 (experiences T2 → intention T3)	Social presence T2 → intention T3	¡0.001	¡0.001	Yes
H2 (experiences T2 → intention T3)	Flow T2 → intention T3	¡0.001	¡0.001	Yes
H2 (experiences T2 → intention T3)	Belonging T2 → intention T3	¡0.001	¡0.001	Yes
H3/H4 (cross-domain)	Intention T3 → activity T4	0.677	0.687	No
H3/H4 (cross-domain)	indirect via presence	0.686	0.687	No
H3/H4 (cross-domain)	indirect via flow	0.684	0.687	No
H3/H4 (cross-domain)	indirect via belonging	0.687	0.687	No
RQ1 (reverse paths, exploratory)	Activity T1 → interaction T2	0.881	0.881	No
RQ1 (reverse paths, exploratory)	Activity T2 → interaction T3	0.102	0.205	No

Table S6: Exploratory equivalence tests for the focal coefficient ($\beta = 0.0155$, bootstrap SE = 0.0373; 90% CI [-0.046, 0.077]). Bounds were chosen after the coefficient was estimated and are reported as a sensitivity range, not as a confirmatory test. The final column gives the power the two one-sided tests would have had at that bound if the true effect were exactly zero.

**SESOI bound**	**TOST** p	**Equivalence established**	**Power if true effect = 0**
±0.05	0.1775	No	0.00
±0.10	0.0117	Yes	0.70
±0.15	0.0002	Yes	0.98

Table S7: The focal coefficient (continuance intention T3 to physical activity T4, adjusting for prior activity, perceived interaction, and demographics) across every specification we estimated. The last row re-estimates from the integer-rounded composite columns stored in the archived data file rather than from the item means specified in the Methods, and is included so that readers can see the scoring rule is not what produces the result.

**Specification**	β	**SE**	**95% CI**	p	n
Primary FIML system (2000 bootstrap)	+0.0155	0.0373	[−0.061, +0.082]	0.677	506
20 chained-equation imputations	+0.0186	0.0382	[−0.057, +0.094]	0.626	506
Complete-case OLS (HC3)	+0.0162	0.0378	[−0.058, +0.090]	0.669	377
log(1+PARS-3) outcome	+0.0220	0.0411	[−0.058, +0.103]	0.592	377
Stabilized IPW for T4 retention	+0.0288	0.0370	[−0.044, +0.101]	0.437	377
Uncorrected T4 retention flags	+0.0135	0.0404	[−0.066, +0.093]	0.738	339
Integer-rounded composites (as filed)	+0.0051	0.0358	[−0.065, +0.075]	0.887	377

Table S8: Multivariable logistic attrition models predicting retention at each wave from baseline constructs and demographics. Predictors are standardized; entries are odds ratios with 95% confidence intervals. Baseline physical activity does not predict retention at any wave; baseline continuance intention does.

**Baseline predictor**	**T2 retention**	**T3 retention**	**T4 retention**
Perceived interaction T1	0.866 [0.676, 1.110]	0.821 [0.660, 1.022]	0.714 [0.565, 0.901]
Social presence T1	0.962 [0.745, 1.242]	0.859 [0.687, 1.074]	0.809 [0.638, 1.026]
Flow T1	1.004 [0.779, 1.295]	1.086 [0.868, 1.358]	1.018 [0.806, 1.286]
Belonging T1	1.013 [0.784, 1.309]	0.959 [0.767, 1.198]	0.984 [0.778, 1.243]
Continuance intention T1	3.212 [2.357, 4.377]	2.870 [2.203, 3.739]	2.968 [2.241, 3.932]
Physical activity T1	0.926 [0.728, 1.178]	0.880 [0.711, 1.088]	0.964 [0.770, 1.207]
Age group	1.107 [0.861, 1.425]	1.097 [0.877, 1.373]	1.180 [0.932, 1.494]
Female	0.975 [0.757, 1.257]	0.923 [0.738, 1.153]	0.998 [0.790, 1.261]
Education	1.000 [0.781, 1.281]	0.834 [0.668, 1.041]	0.830 [0.659, 1.046]
City tier	0.851 [0.658, 1.100]	0.988 [0.789, 1.238]	0.960 [0.759, 1.216]
Years of platform use	0.947 [0.735, 1.221]	0.987 [0.790, 1.233]	0.900 [0.712, 1.137]

Table S9: The perceived-interaction dimensions entered separately in place of the equally weighted index. Each equation controls the prior level of the outcome and the demographic covariates. The composite rows repeat the corresponding index specification for comparison; explanatory power is essentially identical, and in the focal equation none of the three dimensions predicts T4 physical activity.

**Outcome**	**Predictor**	β	**SE**	p	R^2	n
Social presence T2	Modality richness T1	+0.188	0.034	¡0.001	0.541	417
Social presence T2	Bidirectionality T1	+0.135	0.033	¡0.001	0.541	417
Social presence T2	Response immediacy T1	+0.196	0.035	¡0.001	0.541	417
Social presence T2	Composite index T1	+0.294	0.037	¡0.001	0.539	417
Flow T2	Modality richness T1	+0.235	0.029	¡0.001	0.608	417
Flow T2	Bidirectionality T1	+0.226	0.034	¡0.001	0.608	417
Flow T2	Response immediacy T1	+0.173	0.032	¡0.001	0.608	417
Flow T2	Composite index T1	+0.357	0.033	¡0.001	0.605	417
Belonging T2	Modality richness T1	+0.133	0.035	¡0.001	0.495	417
Belonging T2	Bidirectionality T1	+0.147	0.037	¡0.001	0.495	417
Belonging T2	Response immediacy T1	+0.151	0.036	¡0.001	0.495	417
Belonging T2	Composite index T1	+0.243	0.037	¡0.001	0.495	417
Physical activity T4	Modality richness T3	−0.007	0.042	0.868	0.483	377
Physical activity T4	Bidirectionality T3	+0.025	0.036	0.485	0.483	377
Physical activity T4	Response immediacy T3	+0.018	0.037	0.626	0.483	377
Physical activity T4	Continuance intention T3	+0.019	0.036	0.596	0.483	377

Table S10: Baseline values of the focal predictor and related variables split by retention status, in response to a reviewer's request to test range restriction directly. The final column is the ratio of the full screened cohort's standard deviation to that of the retained subsample, which is the factor by which a coefficient estimated on the retained subsample would be attenuated. For continuance intention split by T3 retention — the split that governs the focal equation — that factor is 1.021.

**Split**	**Variable**	n_{in}	M_{in}	SD_{in}	n_{out}	M_{out}	SD_{out}	SD_{full}	**Ratio**
Retained at T3	Continuance intention T1	377	5.054	0.958	129	4.212	0.738	0.978	1.0208
Retained at T3	Perceived interaction T1	377	4.805	0.532	129	4.877	0.547	0.537	1.0080
Retained at T3	Physical activity T1	377	18.897	14.343	129	21.209	16.472	14.932	1.0411
Completed T4	Continuance intention T1	394	5.025	0.966	112	4.185	0.700	0.978	1.0121
Completed T4	Perceived interaction T1	394	4.793	0.536	112	4.929	0.528	0.537	1.0014
Completed T4	Physical activity T1	394	19.213	14.506	112	20.446	16.378	14.932	1.0294

Table S11: Distribution of the PARS-3 score at each wave. Because the score is the product of intensity, duration, and frequency and duration is coded from zero, a respondent in the lowest duration category scores zero whatever their intensity and frequency. Every zero in the data arises this way, so the instrument has a true floor and not merely a skewed lower tail.

**Wave**	n	**Zeros**	**% zero**	**From zero component**	M	**SD**	**Mdn**	**P90**	**Skew**
T1	506	8	1.6	8	19.49	14.93	18.0	48.0	1.30
T2	417	4	1.0	4	19.64	12.63	18.0	36.0	1.29
T3	377	0	0.0	0	19.58	11.37	18.0	36.0	1.05
T4	394	24	6.1	24	20.03	16.08	16.0	40.0	1.36

Table S12: Reconciliation of every quantity reported in the previously submitted version against the rebuilt analysis pipeline. *Match* denotes agreement within rounding; *shift* a numerical update that leaves the conclusion unchanged; *changed* a result whose wording required revision; *new* a quantity added in this revision.

Section	Quantity	As submitted	Rebuilt	Status
3.1	Source records	550	550	match
3.1	T1 median completion time (s)	290	290	match
3.1	Excluded: too fast	19	19	match
3.1	Excluded: long-string >=15	27	27	match
3.1	Excluded: met >1 rule	2	2	match
3.1	Analysis cohort N	506	506	match
3.1	Retention T2 / T3 / T4	417 / 377 / 394	417 / 377 / 394	match
3.1	T4 flag: pre-correction n	not stated in Table 1	356	new
3.1	T4 flag: records reclassified	38 (in running text)	38	match
3.1	T2/T3 flags independently audited	not reported	mismatches T2=0, T3=0	new
3.1	Reclassified records missing T4 duration	not reported	38 of 38	new
3.1	Cronbach alpha range	0.898 – 0.976 (text) / 0.919 – 0.976 (Table 3)	0.919 – 0.976	shift
3.1	McDonald omega range	not reported	0.919 – 0.976	new
3.1	Configural CFI range	0.984 – 1.000	0.989 – 1.000	shift
3.1	Configural RMSEA range	0.000 – 0.045	0.000 – 0.045	match
3.1	Max —dCFI— across constraints	< 0.01	0.0013	match
3.1	T1 standardized loadings	not reported	0.892 – 0.941	new
3.1	Formative facet VIFs	1.00 for each	1.000 – 1.006	shift
3.1	Facet pairwise correlations	not reported	T1: -0.011 / -0.009 / -0.059	new
3.2	Zero-order r: intention T3 – activity T4	0.063	0.068	shift
3.2	r range quoted in Section 3.2	0.272 – 0.511	0.218 – 0.563	shift
3.2	Autoregressive r: activity T3 – T4	0.691	0.691	match
3.2	Activity T4 SD (PARS-3 points)	16.08	16.08	match
Table 5	Interaction T1 → social presence T2	0.220 [0.150, 0.292]	+0.2967 [+0.220, +0.371] p=<0.001	shift
Table 5	Interaction T1 → flow T2	0.324 [0.253, 0.391]	+0.3550 [+0.285, +0.422] p=<0.001	shift
Table 5	Interaction T1 → belonging T2	0.228 [0.159, 0.301]	+0.2452 [+0.175, +0.318] p=<0.001	shift
Table 5	Social presence T2 → intention T3	0.205 [0.131, 0.275]	+0.2025 [+0.133, +0.268] p=<0.001	match
Table 5	Flow T2 → intention T3	0.276 [0.207, 0.344]	+0.3023 [+0.238, +0.360] p=<0.001	shift
Table 5	Belonging T2 → intention T3	0.257 [0.191, 0.322]	+0.2318 [+0.166, +0.296] p=<0.001	shift
Table 5	Activity T3 → activity T4	0.699 [0.621, 0.761]	+0.6976 [+0.623, +0.761] p=<0.001	match
Table 5	Intention T3 → activity T4	-0.0003 [-0.066, 0.068]	+0.0155 [-0.061, +0.082] p=0.677	shift
Table 5	Interaction T3 → activity T4	0.017 [-0.051, 0.092]	+0.0118 [-0.065, +0.087] p=0.763	match
Table 5	Interaction T1 → activity T4	0.020 no interval retained	+0.0175 [-0.060, +0.096] p=0.665	match
Table 5	Activity T1 → interaction T2	0.020 [-0.062, 0.100]	-0.0050 [-0.071, +0.060] p=0.881	shift
Table 5	Activity T2 → interaction T3	0.101 [0.013, 0.189]	+0.0669 [-0.013, +0.146] p=0.102	changed
Table 6	Serial indirect via presence	-0.000 (combined -0.0003)	+0.00093 [-0.00377, +0.00539]	shift
Table 6	Serial indirect via flow	-0.000 (combined -0.0003)	+0.00167 [-0.00665, +0.00952]	shift
Table 6	Serial indirect via belong	-0.000 (combined -0.0003)	+0.00088 [-0.00371, +0.00498]	shift
Table 6	Serial indirect via combined	-0.000 (combined -0.0003)	+0.00348 [-0.01376, +0.01895]	shift
3.6	Focal beta — Complete-case OLS (HC3)	-0.025, p = 0.533	+0.0162, SE = 0.0378, p = 0.669, n = 377	shift
3.6	Focal beta — log(1+PARS-3) outcome	-0.029, p = 0.510	+0.0220, SE = 0.0411, p = 0.592, n = 377	shift
3.6	Focal beta — Stabilized IPW for T4 retention	-0.003, p = 0.947	+0.0288, SE = 0.0370, p = 0.437, n = 377	shift
3.6	Focal beta — Uncorrected T4 retention flags	0.002, SE = 0.041, p = 0.962, n = 339	+0.0135, SE = 0.0404, p = 0.738, n = 339	shift
3.6	Focal beta — 20 imputations	-0.002, p = 0.951	+0.0186, p = 0.626	shift
3.6	Focal beta — integer-rounded composites (as filed)		+0.0051	new
3.6	Person-mean-centered — presence	0.077, p = 0.151	+0.1302, p = 0.0198	changed
3.6	Person-mean-centered — flow	0.137, p = 0.002	+0.1750, p = 0.0005	changed
3.6	Person-mean-centered — belong	0.087, p = 0.122	+0.0895, p = 0.0635	changed
3.5	90% CI for focal coefficient	[-0.055, 0.057]	[-0.0458, +0.0768]	shift
Table 7	TOST p at +/-0.05	0.072, not equivalent	0.1775, not equivalent (power if true zero = 0.00)	shift
Table 7	TOST p at +/-0.10	0.002, equivalent	0.0117, equivalent (power if true zero = 0.70)	shift
Table 7	TOST p at +/-0.15	<0.001, equivalent	0.0002, equivalent (power if true zero = 0.98)	shift
3.5	BIC-approximated BF01	16.84	17.81	shift
3.5	Minimum detectable —beta—	0.146 – 0.169	0.104	changed
3.1	Attrition: baseline activity → T4	OR 1.041 [0.802, 1.350], p = 0.764	OR 0.964 [0.770, 1.207], p = 0.750	shift
3.1	Attrition: baseline intention → T4	OR 3.199 [2.306, 4.438]	OR 2.968 [2.241, 3.932]	shift
3.1	Attrition: education → T4	OR 0.706 [0.541, 0.922], p = 0.011	OR 0.830 [0.659, 1.046], p = 0.115	changed
new	BH-FDR within hypothesis families	not done	no decision changes in any family	new
new	Range restriction on focal predictor	complete-case vs full-cohort M/SD	SD retained 0.958 vs full cohort 0.978; attenuation factor 1.021	new
new	Facets entered separately	not done	all three predict each T2 experience (0.133-0.235, p<.001); equation R2 0.5413	new
new	PARS-3 floor	not reported	zeros: T1 8, T2 4, T3 0, T4 24 (6.1%); every zero from a zero component	new

References

Cheung, G.W.; Rensvold, R.B. Evaluating goodness-of-fit indexes for testing measurement invariance. *Structural Equation Modeling* **2002**, *9*, 233–255.

Chen, F.F. Sensitivity of goodness of fit indexes to lack of measurement invariance. *Structural Equation Modeling* **2007**, *14*, 464–504.
